# Risk Factors for Venous Thromboembolism in 1.3 Million Pregnancies: A Nationwide Prospective Cohort

**DOI:** 10.1371/journal.pone.0096495

**Published:** 2014-05-02

**Authors:** Rie Adser Virkus, Ellen Løkkegaard, Øjvind Lidegaard, Jens Langhoff-Roos, Anne Kristine Nielsen, Kenneth J. Rothman, Thomas Bergholt

**Affiliations:** 1 Department of Obstetrics & Gynecology, Hillerød Hospital, University of Copenhagen, Hillerød, Denmark; 2 Gynecological Clinic, Rigshospitalet, University of Copenhagen, Copenhagen, Denmark; 3 Obstetrical Clinic, Rigshospitalet, University of Copenhagen, Copenhagen, Denmark; 4 Research Triangle Institute, Research Triangle Park, North Carolina, United States of America; Medical Faculty, Otto-von-Guericke University Magdeburg, Medical Faculty, Germany

## Abstract

**Objective:**

To quantify risk factors for venous thromboembolism during pregnancy and the puerperal period.

**Design:**

In a nationwide prospective cohort study we followed pregnant and puerperal women in Denmark from 1995 to 2009 for venous thromboembolism. Information on risk factors and confounders was retrieved from national registries. The diagnosis of venous thromboembolism was confirmed through medical charts. We calculated adjusted incidence rates per 10,000 women years and used Poisson regression to estimate effects during pregnancy and the puerperal period.

**Results:**

We studied 1,297,037 pregnancies and related puerperal periods, during which there were 748 venous thromboembolisms. The incidence rate for venous thromboembolism during a pregnancy with and without hospitalization for hyperemesis was 15.2/10,000 yr and 6.3/10,000 yr, respectively, (adjusted rate ratio: 2.5 (95%-confidence interval; 1.4–4.5)). The incidence rate among women with multiple pregnancies was 18.2/10,000 yr and 6.3/10,000 yr in singletons (adjusted rate ratio: 2.8 (1.9–4.2)). Increased risk was found with hospitalization during pregnancy or the puerperal period with incidence rates of 42.1/10.000 and 54.7/10.000, respectively, (rate ratios: 12.2 (8.7–17) and 5.9 (4.0–8.8)). Women hospitalized with infections during pregnancy had incidence rates of 25.9/10,000 yr and 29.3/10,000 yr during pregnancy and the puerperal period, respectively, and of 62.7/10,000 yr if hospitalized with infection in the puerperal period. Puerperal venous thromboembolism was associated with hospitalization for preeclampsia and intrauterine growth restriction/fetal death with incidence rates of 45.8/10,000 yr and 18.3/10,000 yr, respectively (rate ratio: 5.0 (3.1–7.8) and 1.9 (0.9–4.4)). Additionally puerperal venous thromboembolism was associated with obesity, elective and acute caesarean sections and major postpartum bleeding with incidence rates of 25.5/10,000 yr, 23.2/10,000 yr, 34.0/10,000 yr and 20.3/10,000 yr, respectively (rate ratios 1.7 (1.1–2.7), 2.1 (1.4–3.1), 3.0 (2.3–4.0) and 1.4 (1.0–2.1)).

**Conclusions:**

Important risk factors for venous thromboembolism during pregnancy or the puerperal period were hospitalization, infection, hyperemesis, multiple pregnancies, preeclampsia, obesity, caesarean section, major postpartum bleeding, and intrauterine growth restriction or fetal death.

## Introduction

In the western world, venous thromboembolism in pregnancy and the puerperal period has been either the most common cause of maternal death [1]–[3] or ranked closely behind sepsis and preeclampsia/eclampsia. The risk of venous thromboembolic events has been reported to be in the range from 7 to 25 per 10,000 pregnancies and is highest around delivery, when the risk is more than 20-fold greater than that of non-pregnant women [4]–[14], [14]–[16]. Several risk factors have been identified, but the literature is inconsistent in the quantification of these, in part because prophylactic anticoagulation treatment has not been taken into account [17]–[22]. The purpose of this study was to identify and quantify various risk factors for venous thromboembolism during pregnancy and the puerperal period.

## Methods and Materials

We conducted a retrospective cohort study that included most pregnancies in Denmark from 1995 through 2009. The cohort was established by linking information from six national health registries.

### Data sources

The *Central Person Registry* includes a 10-digit personal identification number given to all Danish citizens at birth or immigration. The number is used in all public registries, thus allowing reliable linkage. The *National Registry of Patients*, established in 1977, collects surgical codes and discharge diagnoses classified according to the World Health Organization's International Classification of Diseases (Version ICD-8 until end of 1993 and version ICD-10 from 1994) (Table 1). The *Danish Cause of Death Registry* collects diagnoses of all cases of death in Denmark, classified like the diseases in National Registry of Patients. *Statistics of Denmark* includes annually updated information on length of schooling and any on-going or completed education. The *National Registry of Medical Products Statistics* capture all redeemed prescriptions to Danish citizens since 1995 by date, Anatomical-Therapeutic-Chemical codes and the amount of medicine prescribed in daily doses. The *Danish Birth Registry* was established 1973 and includes information of all births.

**Table 1:** Definition of exposure variables, confounders and outcome variables from various registries (The National Registry of Patients, The National Registry of medical Products Statistics, The Danish Birth Registry, Statistics of Denmark and Cause of Death Registry) by International Classification of Diseases (ICD), operation and Anatomical Therapeutic Chemical (ATC) codes.

Exposure-variables	Registry^a^	ICD-8/10 diagnoses- or operation codes or ATC-code
Hyperemesis	1	O210-219
Polyhydramnios	1	O40
Preeclampsia	1	O140-149 and O150-159
Infection based at antibiotic treatment	2	MJ01
Infection based on discharge diagnoses	1	O230-239, O411, O740, O752-753, O859-864, O868, O890A, O910-912, O980-989, K35-37, N10, N12 and N30
Plurality, BMI and smoking status^b^	3	
Caesarean section, acute or elective	3	KMCA
Operations in the puerperal period	1	KMWC, KMBA, KMBB10, KMBW96A
Postpartum major bleeding	1	O902, O081, O670-679, O720-723 and OQA0 (treatment code)
Hospitalization in days	1	Cumulative number of days of in hospitalization
Preeclampsia or a hypertensive disorder	1	O100-159
Infection^c^	1	O230-239, O411, O740, O752-753, O859-864, O868, O890A, O910-912, O980-989, K35-37, N10, N12 and N30
Intrauterine growth restriction or fetal death^c^	1	O363-365 and O40
Bleeding episode during pregnancy, placenta previa or abruptio placentae^c^	1	O200-209 and O440-469
Threatening preterm labor or preterm premature rupture of membranes^c^	1	O420, O422, O424, O429 and O472
Hyperemesis^c^	1	O210-219 and O240-249
**Potential confounders**		
Age, calendar-year and parity	3	
Educational status	4	
Thrombophilia	1	D685 and D686
Anticoagulative prophylactic treatment	2	MB01AA, MB01AB, MB01AC, MB01AE and MB01AX
Diabetes mellitus	1	E100-149
Inflammatory bowel disease	1	K500-519
Other inflammatory or rheumatoid disease	1	N040-049, M05-07, M13 and M30-36
Assisted Reproductive Technology	2	MG03GA and MG03GB
**Outcome variable**		
VTE (ICD-8 till 1993)	1	450, 451.00, 451.08, 451.99, 452, 453.02, 631, 634.99, 671.01, 671.01, 671.08, 671.09 and 673
VTE (ICD-10 from 1994)	1 and 5	I26, I676, I80.1, I80.2, I80.3, I81, I82.2, I82.3, I82.8, I82.9, O08.7, O08.7A-F, O22, O22.3, O22.5, O22.5 A, O22.8, O22.9, O87, O87.1, O87.1A, O87.3, O87.3A, O87.8, O87.9, O88, O88.0, O88.0A, O88.0B, O88.1, O88.1A, O88.1B, O88.2 and O88.2 A

a 1. The National Registry of Patients, 2. The National Registry of medical Products Statistics, 3. The Danish Birth Registry (includes births after 22 weeks), 4. Statistics of Denmark, 5. The Cause of Death Registry.

b Routine recording of BMI started in 2004. Smoking status was recorded as a dichotomous variable and BMI in 5 categories: BMI under 18.5, BMI between 18.5–24.9, 24–29.9, 30–35 and BMI more than 35.

c Hospitalization during pregnancy, if admitted more than one day

### Study population

All Danish women 15–49 years old during the period January 1, 1995 through December 31, 2009 were eligible, provided that they had no history of venous thromboembolism, other cardiovascular disease, or cancer. A woman's follow-up was censored at death, emigration, diagnosis of cancer, or diagnosis of any cardiovascular disease, including venous thromboembolism.

Pregnant women in this population were identified in the National Registry of Patients. The puerperal period comprised the 12 weeks after the delivery or 8 weeks after a pregnancy that was terminated early.

### Identification of exposures

We considered several potential risk factors for venous thromboembolism in pregnancy and the puerperal period (Table 1). Smoking, body mass index, and multiple pregnancies were recorded once in the beginning of each pregnancy as indicator variables. Hospitalization during pregnancy or the puerperal period, with a primary discharge diagnosis of hyperemesis, polyhydramnios, preeclampsia, infection was evaluated as time-dependent variables. Infection primary discharge diagnoses comprised infections of kidney, bladder, urethra, of other or unspecified parts of urinary tract, infections of the genital tract or unspecified genitourinary tract infection in pregnancy, infection of amniotic sac and membranes, aspiration pneumonitis due to anesthesia during labor and delivery, pyrexia or other infection during labor not elsewhere classified, infection of obstetric surgical wound, other infection of genital tract or urinary tract following delivery, pyrexia of unknown origin or other unspecified puerperal infections following delivery, pulmonary complications of anesthesia during the puerperium, infection of nipple or abscess of breast, non-purulent mastitis associated with pregnancy, the puerperium and lactation, tuberculosis, syphilis, gonorrhea, viral hepatitis or other viral disease, protozoal disease, human immunodeficiency virus or other or unspecified maternal infectious and parasitic diseases complicating pregnancy, childbirth and the puerperium, acute appendicitis, other or unspecified appendicitis, acute tubulo-interstitial nephritis, tubulo-interstitial nephritis and tubulo-interstitial nephritis, not specified as acute or chronic and cystitis as a non-specific pregnancy ICD-code.

Filling a prescription for an antibiotic treatment at a pharmacy was also included as a time-dependent variable.

Polyhydramnios was considered a risk factor related only during pregnancy, whereas mode of delivery and postpartum bleeding were indicator variables considered as possible risk factors for puerperal venous thromboembolism.

Hospitalizations during pregnancy were recorded in the *National Patient Registry* and include information on cause of admission. We included hospitalizations for more than one day. To capture the possible effect of immobilization itself and distinguish that from other thrombogenic causes, women were classified according to presumptively thrombogenic diagnoses, specifically preeclampsia, hyperemesis, infection, fetal complication, (which included intrauterine growth restriction or intrauterine fetal death) and bleeding (which included placental abruption or placenta previa). We also classified women by an indicator of presumptively non-thrombogenic diagnoses (threatening preterm labor or preterm premature rupture of membranes). To summarize information on length of hospitalization, we created a variable that summed all hospitalization days for all stays in hospital during the pregnancy or puerperal period. During pregnancy this variable summarized days of hospitalization from the second day of admission until either a thromboembolic event or delivery. In the puerperal period we used another variable for days of hospitalization, constructed the same way. We categorized these variables as follows: 1–2 days, 3–7 days, 8–14 days, and more than 14 days.

We controlled for factors associated with occurrence of venous thromboembolism; these included maternal age (5-yr categories), parity (1, 2, 3, 4+), education (four levels) and diagnosis of thrombophilia, diabetes mellitus, inflammatory and rheumatoid diseases and inflammatory bowel disease at some time before or during pregnancy, the latter all coded as indicator variables. We included as time-dependent variables the filling of a prescription for anticoagulation treatment during pregnancy or the puerperal period and receiving ovarian stimulation therapy for assisted reproduction, up to 12 weeks before pregnancy.

### Identification of outcome

We identified the first occurrence of venous thromboembolism during pregnancy or the puerperal period from the *National Registry of patients* and the *Cause of Deaths Registry*. We included deep venous thrombosis of the lower or upper extremities, pulmonary embolism, cerebral venous thrombosis, portal vein thrombosis, vena cava thrombosis and ovarian vein thrombosis (Table 1).The diagnoses were validated by review of the medical records [23]The diagnosis of venous thromboembolism was considered validated if there was at least one relevant confirmatory diagnostic test result from ultrasonography, venography, ventilation-perfusion lung scan, computer-tomography or a magnetic resonance scan, or if the woman received anticoagulation therapy in therapeutic doses for the rest of the pregnancy or puerperal period, or for at least three months. The exact date for the confirmatory diagnostic test or the beginning of treatment was noted and used as the “*validated date for the diagnosis*”. If the exact date was not found, the admission date was used (7.2%).

Ethical approval is not needed for registry-based studies in Denmark, but the study was approved by the Danish Data Protection Agency (J.no: 2011-41-5619). Patient records and information were anonymized and de-identified before analysis.

### Analyses

We first conducted stratified analyses to examine the relation between each exposure and the main confounders and calculated incidence rates adjusted for confounding from the Poisson regression model. In subsequent analyses we fit separate Poisson regression models for each of the two risk periods, pregnancy and the puerperal period, to control simultaneously for all measured potential confounders (age, educational status, calendar year, parity, thrombophilia, anticoagulative medication, chronic medical diseases and assisted reproductive therapy). Information on smoking, BMI, plurality and mode of delivery was not available for pregnancies that ended before week 22. To deal with the missing data on BMI before 2004, the analyses on this variable were restricted to the years when BMI was available. We redid the primary analyses using Generalized Estimating Equations (GEE), which takes into account the correlated outcomes when the data contain more than one birth from some women. Using GEE, the results remained identical through two decimal places.

The data were analyzed using SAS statistical software version 9.1.

## Results

We identified 1,320,353 pregnancies among 656,300 women, and then excluded pregnancies for which the woman had a diagnosis of venous thromboembolism, cardiovascular disease or cancer before the study period (10,559) or during the study period but before the pregnancy (14,852). After these exclusions, 1,297,037 pregnancies remained. Of these, 901,758 continued through 22 weeks of gestation or more. There were 748 validated cases of venous thromboembolism, of which 433 (57.9%) occurred during pregnancy and 315 (42.1%) during the puerperal period. Of the antenatal cases, 94 (21.7%) were diagnosed during the first trimester, 144 (33.3%) during the second, and 195 (44.0%) during the third trimester.

The incidence rates per 10,000 woman-years at risk were 3.9, 5.2 and 10.5 for each trimester, respectively. Table 2 shows the incidence rates of venous thromboembolism according to values of the primary possible confounding factors. There were only minor differences between unadjusted and age-adjusted rate ratios for the various exposure variables during pregnancy and in the puerperal period, indicating that age was generally not an important confounder (Tables 3 and 4). With further adjustment for the other potential confounding factors, effect estimates declined, indicating that there was some confounding effect from the factors other than age.

**Table 2:** Incidence rate of venous thromboembolism (VTE) among Danish women during pregnancy and the puerperal period according to age, education, parity and other risk factors during the period 1995–2009.

Risk factor	Pregnancy				Puerperal period		
	VTE (n)	Women years	%	Incidence rate^a^	VTE (n)	Women years	Incidence rate^a^
**Age**							
15–19 yrs.	9	17,583	2.5	5.1	5	5,564	9.0
20–24 yrs.	56	96,372	13.7	5.8	40	28,986	13.8
25–29 yrs.	131	247,767	35.3	5.3	96	76,317	12.6
30–34 yrs.	155	234,021	33.3	6.6	98	79,694	12.3
35–39 yrs.	71	91,381	13.0	7.8	59	34,242	17.2
40–44 yrs.	10	14,854	2.1	6.7	16	6,565	24.4
45–49 yrs.	1	603	0.1	16.6	1	323	31.0
**Education** b							
Elementary school completed	91	132,880	18.9	6.8	93	45,307	20.5
High school on-going or completed	38	54,099	7.7	7.0	16	18,232	8.8
High school plus middle on-going or completed	169	259,571	36.9	6.5	105	85,205	12.3
High school plus long on-going or completed	129	235,075	33.5	5.5	92	76,121	12.1
Unknown	6	20,957	3.0	2.9	9	6,827	13.2
**Parity**							
1	228	276,255	39.3	8.3	132	85,967	15.4
2	102	226,085	32.2	4.5	89	70,290	12.7
3	44	85,505	12.2	5.1	36	26,609	13.5
4+	24	35,907	5.1	6.7	24	11,146	21.5
Unknown^c^	35	78,829	11.2	4.4	12	8,661	9.0
**Other risk factor**							
Thrombophilia	7	357	0.1	196.3	3	177	169.2
No thrombophilia	426	702,225	99.9	6.1	312	231,514	13.5
Anticoagulation	10	856	0.1	116.8	6	545	110.2
No anticoagulation	423	701,726	99.9	6.0	309	231,147	13.4
Inflammatory disease	11	10,611	1.5	10.4	11	3,552	31.0
No inflammatory disease	422	691,970	98.5	6.1	304	228,139	13.3
Inflammatory bowel disease	8	7,232	1.0	11.1	5	2,402	20.8
No inflammatory bowel disease	425	695,350	99.0	6.1	310	229,289	13.5
Diabetes Mellitus	3	5,903	0.8	5.1	9	2,043	44.0
No Diabetes Mellitus	430	696,679	99.2	6.2	306	229,648	13.3
Assisted reproductive therapy	48	31,152	4.4	15.4	15	10,104	14.8
No assisted reproductive therapy	385	671,429	95.6	5.7	300	221,587	13.5

a Incidence rate is number per 10,000 women-years at risk

b In Denmark middle education is defined as 4 years of education after high school, and long education as 5 to 6 years of education after high school

c Information necessary is not available for pregnancies that ended before week 22.

**Table 3:** Prevalence, incidence rate, age-adjusted rate ratio with 95% confidence interval and confounder-adjusted rate ratios of venous thromboembolism (VTE) **during pregnancy** according to different risk factors.

Risk factor	%	VTE (n)	Incidence rate^a^	Rate ratios (95% CI)		
				Crude	Age adjusted	Adjusted^b^
**Smoking** c						
Non-smoker	77.1	318	6.3	1	1 (ref)	1 (ref)
Smoker	19.0	73	5.9	1.0	0.9 (0.7–1.2)	0.9 (0.7–1.2)
Unknown	3.8	26	11.4	1.7	1.7 (1.1–2.5)	1.8 (1.2–2.7)
**Body-mass index (kg/m2)** c						
<18.5	3.9	6	6.1	0.9	0.9 (0.4–2.0)	0.9 (0.4–2.0)
18.5–24.9	58.5	102	7.0	1	1 (ref)	1 (ref)
25–29.9	19.4	49	9.8	1.5	1.5 (1.0–2.1)	1.4 (1.0–2.0)
30–34.9	7.2	13	6.8	1.0	1.1 (0.6–1.9)	1.0 (0.6–1.8)
>35	3.8	5	4.9	0.8	0.8 (0.3–1.9)	0.7 (0.3–1.8)
Unknown	7.2	13	7.3	1.0	1.0 (0.6–1.8)	1.1 (0.6–2.0)
**Hyperemesis**						
No hyperemesis	99.0	422	6.3	1	1 (ref)	1(ref)
During pregnancy	1.0	11	15.2	2.7	2.7 (1.5–4.9)	2.5 (1.4–4.5)
**Plurality** c						
Singleton pregnancy	98.0	351	6.3	1	1 (ref)	1 (ref)
Multiple pregnancy	2.0	28	18.2	4.0	3.9 (2.6–5.7)	2.8 (1.9–4.2)
**Infection**						
No antibiotic treatment	83.2	314	5.7	1	1 (ref)	1 (ref)
Antibiotic treatment	16.8	119	9.8	1.9	1.9 (1.5–2.3)	1.8 (1.5–2.3)
No infection discharge diagnoses	99.2	416	6.2	1.1	1 (ref)	1 (ref)
Infection discharge diagnoses	0.8	17	25.9	5.1	5.2 (3.2–8.4)	4.3 (2.7–7.1)
**Hospitalization**						
No	82.7	196	3.5	1	1 (ref)	1 (ref)
1–2 days	3.1	78	35.1	10.7	10.7 (8.2–14.0)	10.3 (7.9–13.4)
3–7 days	1.4	41	42.1	12.6	12.6 (9.0–17.7)	12.2 (8.7–17.0)
8–14 days	1.0	10	13.7	4.2	4.2 (2.2–7.9)	4.0 (2.0–7.3)
>14 days	11.9	108	11.4	3.8	3.8 (3.0–4.8)	3.3 (2,6–4,2)
**Preeclampsia**						
No preeclampsia	99.5	414	6.4	1	1 (ref)	1 (ref)
Preeclampsia	0.5	3	7.1	1.3	1.3 (0.4–4.1)	1.2 (0.4–3.6)

a Adjusted incidence per 10,000 women-years at risk.

b Rate ratio adjusted for age, calendar-year, educational status, thrombophilia, anticoagulation treatment, medical diseases, assisted reproductive treatment, and parity.

c Information necessary for the full analysis is not available for pregnancies that ended before week 22 (7% of the risk time).

**Table 4:** Incidence rate, age-adjusted and confounder-adjusted rate ratios for venous thromboembolism (VTE) during **the puerperal period** according to different risk factors.

Risk factor	%	VTE (n)	Incidence rate^a^	Rate ratios (95% CI)		
				Crude	Age adjusted	Adjusted^b^
**Smoking** c						
Non-smoker	77.0	207	13.7	1	1 (ref)	1 (ref)
Smoker	19.2	70	16.1	1.4	1.4 (1.0–1.8)	1.2 (0.9–1.6)
Unknown	3.8	16	19.9	1.6	1.6 (0.9–2.6)	1.5 (0.9–2.4)
**Body-mass index (kg/m2)** c						
<18.5	3.9	4	12.2	1.3	1.3 (0.5–3.6)	1.2 (0.4–3.4)
18.5–24.9	57.6	47	9.9	1	1 (ref)	1 (ref)
25–29.9	19.0	27	16.8	1.7	1.7 (1.1–2.8)	1.7 (1.1–2.7)
30–34.9	7.1	13	20.7	2.3	2.2 (1.2–4.1)	2.1 (1.1–3.9)
>35	3.7	12	34.8	4.0	4.0 (2.1–7.5)	3.5 (1.8–6.7)
Unknown	8.7	14	17.7	2.0	1.9 (1.1–3.5)	1.8 (1.0–3.3)
**Plurality** c						
Singleton pregnancy	97.9	264	14.7	1	1 (ref)	1 (ref)
Multiple pregnancy	2.1	8	18.9	1.4	1.4 (0.7–2.72)	1.3 (0.6–2.6)
**Infection during pregnancy**						
No antibiotic treatment	70.7	204	12.5	1	1 (ref)	1 (ref)
Antibiotic treatment	29.3	111	16.1	1.3	1.3 (1.0–1.7)	1.3 (1.0–1.6)
No infection discharge diagnoses	97.5	296	13.1	1	1 (ref)	1 (ref)
Infection discharge diagnoses	2.5	19	29.3	2.5	2.6 (1.6–4.1)	2.4 (1.5–3.8)
**Infection in puerperal period**						
No antibiotic treatment	90.2	274	13.1	1	1 (ref)	1 (ref)
Antibiotic treatment	9.8	41	17.8	1.4	1.4 (1.0–1.9)	1.4 (1.0–1.9)
No infection discharge diagnoses	99.6	308	13.4	1	1 (ref)	1 (ref)
Infection discharge diagnoses	0.4	7	62.7	5.3	5.2 (2.5–11.1)	5.0 (2.4–10.6)
**Mode of delivery** c						
Vaginal	89.0	188	11.1	1	1 (ref)	1 (ref)
Elective cesarean section	6.4	30	23.2	2.2	2.1 (1.5–3.2)	2.1 (1.4–3.1)
Acute cesarean section	11.0	75	34.0	3.2	3.2 (2.4–4.2)	3.0 (2.3–4.0)
**Postpartum bleeding**						
No bleeding	93.8	287	13.2	1	1 (ref)	1 (ref)
Major bleeding	6.2	28	20.4	1.5	1.4 (1.0–2.1)	1.4 (1.0–2.1)
**Hospitalization**						
No	62.1	137	9.8	1	1 (ref)	1 (ref)
1–2 days	12.0	40	14.2	1.5	1.4 (1.0–2.0)	1.5 (1.0–2.1)
3–7 days	19.3	62	13.3	1.5	1.5 (1.1–2.0)	1.4 (1.1–2.0)
8–14 days	4.5	45	38.7	4.5	4.5 (3.2–6.3)	4.2 (3.0–6.0)
>14 days	2.2	31	54.7	6.7	6.7 (4.5–9.9)	5.9 (4.0–8.8)

a Adjusted incidence per 10,000 women-years at risk.

b Rate ratio adjusted for age, calendar-year, educational status, thrombophilia, anticoagulation treatment, medical diseases, assisted reproductive treatment, and parity.

c Information necessary for the full analysis is not available for pregnancies that ended before week 22 (7% of the risk time).

### Risk factors for venous thromboembolism in pregnancy (Table 3)

Current smokers had no apparent increased risk of venous thromboembolism relative to non-smokers, with a rate ratio of 0.9 (95% CI, 0.7 to1.2). Women whose information on smoking was missing had a rate ratio of venous thromboembolism of 1.8 (95% CI 1.2–2.7) compared with non-smokers. Hyperemesis was associated with an increased risk of venous thromboembolism in pregnancy, with a rate ratio of 2.5 (95% CI, 1.4 to 4.5). Women with a BMI between 25 and 29.9 had a rate ratio of venous thromboembolism of 1.4 (95% CI, 1.0 to 2.0), relative to women with a BMI of 18.5 to 24.9, whereas values of BMI greater than 25 were not associated with notably increased risk for venous thromboembolism. Multiple pregnancies were associated with a rate ratio for venous thromboembolism of 2.8 (95% CI, 1.9 to 4.2) compared with singleton pregnancies.

Receiving antibiotic treatment for infection during pregnancy was associated with a rate ratio of venous thromboembolism of 1.8 (95% CI, 1.5 to 2.3). For women who had a discharge diagnosis of infection during pregnancy the rate ratio was 4.3 (95% CI, 2.7 to 7.1) adjusted for all confounders. Hospitalization 3–7 days during pregnancy increased the risk for venous thromboembolism 12.2 fold (95% CI, 8.7–17.0). Shorter and longer stays also increased the risk for venous thromboembolism, although less so. The risk associated with polyhydramnios was not assessed as there were few women with a diagnosis of polyhydramnios and only one case of venous thromboembolism among these women.

### Risk factors for venous thromboembolism in the puerperal period (Table 4)

Smoking at the beginning of pregnancy was only weakly associated with risk of venous thromboembolism during the puerperal period. Treatment with antibiotics during pregnancy was associated with a rate ratio of venous thromboembolism in the puerperal period of 1.3 (95% CI, 1.0 to 1.6); the corresponding figure for women who were treated during the puerperal period was 1.4 (95% CI, 1.0 to 1.9). Women with a hospital diagnosis of infection in pregnancy or the puerperal period had a rate ratio of venous thromboembolism of 2.4 (95% CI, 1.5 to 3.8) and 5.0 (95% CI, 2.4 to 10.6), respectively. There was only a weak association between risk of venous thromboembolism and being hospitalized less than a week in the puerperal period, whereas women with a hospitalization of 8–14 days or more than 14 days had a rate ratio of 4.2 (95% CI, 3.0 to 6.0) and 5.9 (95% CI, 4.0 to 8.8), respectively. Although there was a strong association between a multiple pregnancy and risk of venous thromboembolism during pregnancy, the rate ratio in the puerperal period after carrying more than one fetus was only 1.3 (95% CI, 0.6 to 2.6).

Compared with women of normal BMI, women with BMI between 25 and 29.9, 30 and 34.9 and 35+ had a rate ratio of puerperal venous thromboembolism of 1.7 (95% CI, 1.1 to 2.7), 2.1 (95% CI, 1.1 to 3.9), and 3.5 (95% CI, 1.8 to 6.7), respectively. Women undergoing elective caesarean sections or emergency caesarean sections had a rate ratio of puerperal venous thromboembolism of 2.1 (95% CI, 1.4 to 3.1) and 3.0 (95% CI, 2.3 to 4.0), respectively, compared with women giving birth vaginally. Women diagnosed with major postpartum bleeding had a rate ratio of puerperal venous thromboembolism of 1.4 (95% CI, 1.0 to 2.1).

We estimated the risk of puerperal venous thromboembolism for women hospitalized more than 1 day during pregnancy according to selected causes of admission to hospital (Table 5). Admission for preeclampsia and a bleeding episode including abruption placenta and placenta previa during pregnancy had a strong thrombogenic effect that persisted in the puerperal period. The rate ratio for preeclampsia was 5.0 (95% CI, 3.1 to 7.8) and a bleeding episode 2.1 (95% CI, 1.1 to 4.3). We found only a modestly elevated increased risk of puerperal thromboembolism when the primary cause of admission during pregnancy was threatened preterm labor (1.6 (0.7–4.0)), whereas when this diagnosis was combined with one or more other diagnoses it was considerably increased (3.4 (2.0–6.0)). When all the above analyses were repeated counting the non-validated cases of venous thromboembolism in addition to validated events, we obtained substantially the same results as reported above.

**Table 5:** Incidence rate, age-adjusted and confounder-adjusted rate ratio of venous thromboembolism (VTE) **during the puerperal period** for women hospitalized more than one day during pregnancy according to six various causes of admission.

Admission for risk factor	VTE (n)	Incidence rate^a^	Rate ratios (95% CI)		
			Crude	Age adjusted	Adjusted^b^
**Hyperemesis**					
No admission	140	9.8	1	1 (ref)	1 (ref)
Hyperemesis	4	15.5	1.6	1.6 (0.6–4.1)	1.2 (0.4–3.3)
Hyperemesis and other^c^	8	29.1	3.0	2.9 (1.4–5.9)	2.0 (0.9–4.4)
Other^c^	163	19.4	2.0	2.0 (1.6–2.5)	1.9 (1.6–2.4)
**Infection**					
No admission	140	9.8	1	1 (ref)	1 (ref)
Infection	74	12.7	1.3	1.3 (1.0–1.7)	1.3 (0.9–1.7)
Infection and other^c^	42	36.1	3.7	3.7 (2.6–5.2)	3.4 (2.4–4.8)
Other^c^	59	30.7	3.1	3.1 (2.3–4.2)	2.7 (2.0–3.8)
**Intrauterine fetal cause** d					
No admission	140	9.8	1	1 (ref)	1 (ref)
Intrauterine fetal cause	6	20.0	2.0	2.0 (0.9–4.6)	1.9 (0.9–4.4)
Intrauterine fetal cause and other^c^	20	61.6	6.3	6.3 (3.9–10.0)	5.6 (3.5–9.0)
Other^c^	149	18.0	1.8	1.7 (1.3–2.3)	1.8 (1.4–2.2)
**Bleeding** e					
No admission	140	9.8	1	1 (ref)	1 (ref)
Bleeding	9	21.3	2.2	2.1 (1.1–4.2)	2.1 (1.1–4.3)
Bleeding and other^c^	15	36.9	3.8	3.7 (2.2–6.4)	3.4 (2.0–5.8)
Other^c^	151	18.7	1.9	1.9 (1.5–2.4)	1.8 (1.5–2.3)
**Threatened preterm labor** f					
No admission	140	9.8	1	1 (ref)	1 (ref)
Threatened preterm labor	5	16.5	1.7	1.7 (0.7–4.1)	1.6 (0.7–4.0)
Threatened preterm and other^c^	14	37.5	3.8	3.8 (2.1–6.6)	3.4 (2.0–6.0)
Other^c^	156	18.9	1.9	1.9 (1.5–2.4)	1.9 (1.5–2.3)
**Preeclampsia**					
No admission	140	9.8	1	1 (ref)	1 (ref)
Preeclampsia	21	49.4	5.0	5.0 (3.2–7.9)	5.0 (3.1–7.8)
Preeclampsia and other^c^	28	70.2	7.1	7.1 (4.7–10.6)	6.4 (4.3–9.8)
Other^c^	126	15.6	1.6	1.6 (1.3–2.0)	1.5 (1.2–2.0)

a Incidence per 10,000 women-years at risk.

b Rate ratio adjusted for age, calendar-year, educational status, thrombophilia, anticoagulation treatment, medical diseases, assisted reproductive treatment.

c Hyperemesis, infection, intrauterine growth restriction, intrauterine fetal death, bleeding episode, abruptio placentae, placenta previa, threatened preterm labor, preterm premature rupture of membranes and preeclampsia.

d Intrauterine growth restriction or intrauterine fetal death,

e Bleeding episode in pregnancy, abruptio placentae or placenta previa.

f Threatened preterm labor, preterm premature rupture of membranes.

## Discussion

We found that the most important risk factors for venous thromboembolism during pregnancy or the puerperal period were maternal age, hyperemesis, multiple pregnancies, infection, hospitalization, preeclampsia, adiposity, caesarean section, and major postpartum bleeding.

As exposure for the majority of study variables was ascertained in healthy women before the outcome of interest, we believe there is little selection bias. Any data source contains errors. Errors in the registry data, however, should be non-differentially related to other study variables. Non differential misclassification would tend to bias the estimates of effect toward the null hypothesis. Misspecification of the duration of the postnatal time window at increased risk from events occurring during pregnancy would also result in a bias toward the null [24]. Thus, the implication of most errors in the data or in our assumptions would be that our estimates are underestimated. Women receiving prophylactic anticoagulation treatment in pregnancy would usually have been diagnosed with thrombophilia or have previously had a venous thromboembolism. Despite our exclusion of women with prior venous thromboembolism, women receiving anticoagulation are at a higher risk for venous thromboembolism than women who are not receiving anticoagulation, and thus the association between this treatment and the risk for venous thromboembolism is presumably confounded by an underlying increased risk for thromboembolism. In Denmark a preconception screening for thrombophilia is not performed routinely. It is only performed on indication as repeated abortions, a child birth with indications of malplacentation, hereditary predisposition to thromboembolism or thromboembolic event. The latter were excluded from the present analyses. Some women might have received anticoagulation treatment from the hospital, typically for a known thrombophilia. Consequently we included as confounding variables a time updated variable from the first date a filled prescriptions for anticoagulation treatment and a diagnosis of thrombophilia. As data are register based we did not have information on any kind of non-pharmacological thromboprophylaxis.

A change in the diagnostic criteria of venous thromboembolism over time was possible; consequently we adjusted for calendar year in all analyses. Virkus et al reported that the validity of diagnoses for venous thromboembolism was not modified by calendar year in a study on the validity of the register-based diagnoses of the venous thromboembolism [23].

Focus on the unhealthful side effects of smoking during pregnancy has increased during the past decade. Consequently, information on smoking during pregnancy may be more likely than ever to be misreported. The fact that women with unknown smoking status had a higher risk for venous thromboembolism during pregnancy and the puerperal period compared with smokers could reflect a disproportionate number of smokers with higher tobacco consumption among women with missing information on smoking. Other studies have found smoking to be a risk factor for venous thromboembolism in pregnancy [8], [13], [17], [20]–[22].

Women with severe preeclampsia usually deliver soon after admission for their preeclampsia, so that their time at risk for venous thromboembolism during pregnancy is brief, but they continue to be at increased risk during the puerperal period, when preeclampsia is an important risk factor for venous thromboembolism. Two previous studies reported preeclampsia to be associated with puerperal venous thromboembolism [8], [10].

Our results showed a strong association between infections and risk for venous thromboembolism, supporting the theory that infections are thrombogenic [25]. We included two definitions of infection in the analyses, one based on filled prescriptions for antibiotics and the other based on in hospitalization. Both variables showed an association with venous thromboembolism. And as the risk increased with increasing severity of infection, it supports the view that infection has a causal role.

A recent study found a rate ratio for venous thromboembolism associated with urinary tract infection of 1.80 (95% CI 1.22–2.67) and with acute respiratory tract infection during pregnancy 1.65 (95% CI 0.94–2.90) based on medical records from British general practitioners [22]. Another recent Danish study that focused on pharmacological risk factors also found increased risk with antibiotics during pregnancy in a register based study [26]. Three studies have found infection in the puerperal period associated with venous thromboembolism [19], [21], [22]. Our finding adds to the evidence that infection is an important risk factor for venous thromboembolism in relation to pregnancy and the puerperal period.

We found hospitalization during pregnancy to be a risk factor for puerperal venous thromboembolism. This risk was influenced by the cause of admission. The highest risks were found with preeclampsia, bleeding episode and also intrauterine growth restriction or intrauterine fetal death especially when combined with other diagnoses. This finding is in accordance with a recent study by Sultan et al., who reported increased risk of venous thromboembolism in cases with intrauterine fetal death [22]. We merged intrauterine growth restriction and intrauterine fetal death because there were few cases and found increased risk comparable with what Sultan et al. reported. No other study investigated this association.

Different time periods and definitions of immobilization may explain the discrepant findings on risk associated with hospitalization in the literature [27], [28]. Hospitalization for a few days in the puerperal period typically reflects a normal delivery or uncomplicated caesarean section. In contrast, admission for a few days during pregnancy could be the result of various adverse conditions. The lower risk of venous thromboembolism with increasing length of hospital stay during pregnancy could reflect an effect of preventive anticoagulation medications or elastic stockings given to women during long hospital stays. Also a long cumulative duration of hospitalization may reflect several shorter hospitalizations as days of hospitalization were summed. A recent study supports increased risk with hospitalization and finds a 4-fold and 12-fold increased risk of venous thromboembolism with hospitalization less than 3 days and more than 3 days, respectively [29]. The study by Jacobsen et al. found 40 times increased risk of venous thromboembolism in the postnatal period among obese women hospitalized during pregnancy [21]. They manually reviewed all the charts and defined immobilization as bed rest for five days or more whereas we used days in hospital. This might explain why we did not find similarly increased risks.

The strong association between multiple pregnancies and venous thromboembolism in pregnancy, but not in the puerperal period was also found in other studies [8], [10], [16], [19], [30], but not in the recent study by Sultan et al. [22]. The association between hyperemesis and venous thromboembolism in pregnancy confirms an earlier report [19], [22]. Postpartum bleeding increased the risk of puerperal venous thromboembolism substantially, in accord with previous studies [2], [19], [21]. Several studies have found an association between BMI in early pregnancy and venous thromboembolism during pregnancy and the puerperal period [18]–[21], [31], [32]. One of these found weight gain during pregnancy of more than 21 kilogram to increase the risk for venous thromboembolism, but only in the puerperal period [10]. Another study showed obesity to be associated with a higher risk of pulmonary embolism than of deep venous thrombosis [20]. The small association between BMI and venous thromboembolism during pregnancy that we found could reflect a more aggressive prophylactic awareness for this category of pregnant women in Denmark. Similar findings were found in the study based on the British general practitioners registry [22].

The strong association between caesarean section and risk of venous thromboembolism is in accordance with previous studies reporting rate ratios from 1.3 to 4.9 [8], [18], [21], [22], [33]. In a Norwegian case-control study from 2008, the authors emphasized that adjusted rate ratios of 1.3 (95% CI; 0.7 to 2.2) and 2.7 (95% CI, 1.8 to 4.1) for elective and acute caesarean section, respectively, could be underestimated, owing to the prophylactic anticoagulation treatment received by most women undergoing caesarean section [21]. Overall, these findings help elucidate characteristics of these women and their pregnancies that may be useful for defining those at high enough risk that they might benefit from preventive anticoagulation measures, given the balance of overall risks as well as costs and benefits for both mother and child.

## Conclusion

We found an increased risk of venous thromboembolism after activation of the inflammatory system, which we assessed by examining filled prescriptions for antibiotics and additionally based on hospitalization discharge diagnoses that indicated infection. We also found that hospitalization itself is associated with an increased risk of thromboembolism, especially when the causes for admission were preeclampsia, bleeding episode and also intrauterine growth restriction or intrauterine fetal death. We confirmed that maternal age, hyperemesis, multiple pregnancies, obesity, and caesarean section are important risk factors for venous thromboembolism during pregnancy or the puerperal period.

### Transparency declaration

EL and RAV affirm that the manuscript is an honest, accurate, and transparent account of the study being reported; no important aspects of the study have been omitted; and there were no discrepancies from the study as planned.

## References

[1] Bodker B, Hvidman L, Weber T, Moller M, Aarre A, et al (2009) Maternal deaths in denmark 2002–2006. Acta Obstet Gynecol Scand 88: 556–562. 19353337 10.1080/00016340902897992

[2] Morris JM, Algert CS, Roberts CL (2010) Incidence and risk factors for pulmonary embolism in the postpartum period. J Thromb Haemost 8: 998–1003. 20128859 10.1111/j.1538-7836.2010.03794.x

[3] Cantwell R, Clutton-Brock T, Cooper G, Dawson A, Drife J, et al (2011) Saving mothers' lives: Reviewing maternal deaths to make motherhood safer: 2006-2008. the eighth report of the confidential enquiries into maternal deaths in the united kingdom. BJOG 118 Suppl 11–203. 10.1111/j.1471-0528.2010.02847.x 21356004

[4] Brown HL, Hiett AK (1996) Deep venous thrombosis and pulmonary embolism. Clin Obstet Gynecol 39: 87–100. 8635310 10.1097/00003081-199603000-00009

[5] McColl MD, Ramsay JE, Tait RC, Walker ID, McCall F, et al (1997) Risk factors for pregnancy associated venous thromboembolism. Thromb Haemost 78: 1183–1188. 9364982

[6] Andersen BS, Steffensen FH, Sorensen HT, Nielsen GL, Olsen J (1998) The cumulative incidence of venous thromboembolism during pregnancy and puerperium—an 11 year danish population-based study of 63,300 pregnancies. Acta Obstet Gynecol Scand 77: 170–173. 9512321

[7] Gherman RB, Goodwin TM, Leung B, Byrne JD, Hethumumi R, et al (1999) Incidence, clinical characteristics, and timing of objectively diagnosed venous thromboembolism during pregnancy. Obstet Gynecol 94: 730–734. 10546719 10.1016/s0029-7844(99)00426-3

[8] Lindqvist P, Dahlback B, Marsal K (1999) Thrombotic risk during pregnancy: A population study. Obstet Gynecol 94: 595–599. 10511366 10.1016/s0029-7844(99)00308-7

[9] Heit JA, Kobbervig CE, James AH, Petterson TM, Bailey KR, et al (2005) Trends in the incidence of venous thromboembolism during pregnancy or postpartum: A 30-year population-based study. Ann Intern Med 143: 697–706. 16287790 10.7326/0003-4819-143-10-200511150-00006

[10] Jacobsen AF, Skjeldestad FE, Sandset PM (2008) Incidence and risk patterns of venous thromboembolism in pregnancy and puerperium—a register-based case-control study. Am J Obstet Gynecol 198: 233.e1–233.e7. 17997389 10.1016/j.ajog.2007.08.041

[11] Pomp ER, Lenselink AM, Rosendaal FR, Doggen CJ (2008) Pregnancy, the postpartum period and prothrombotic defects: Risk of venous thrombosis in the MEGA study. J Thromb Haemost 6: 632–637. 18248600 10.1111/j.1538-7836.2008.02921.x

[12] Martineau M, Nelson-Piercy C (2009) Venous thromboembolic disease and pregnancy. Postgrad Med J 85: 489–494. 19734517 10.1136/pgmj.2009.079822

[13] James AH (2010) Pregnancy and thrombotic risk. Crit Care Med 38: S57–63. 20083915 10.1097/CCM.0b013e3181c9e2bb

[14] Virkus RA, Lokkegaard EC, Bergholt T, Mogensen U, Langhoff-Roos J, et al (2011) Venous thromboembolism in pregnant and puerperal women in denmark 1995–2005. A national cohort study. Thromb Haemost 106: 304–309. 21713323 10.1160/TH10-12-0823

[15] Jackson E, Curtis KM, Gaffield ME (2011) Risk of venous thromboembolism during the postpartum period: A systematic review. Obstet Gynecol 117: 691–703. 21343773 10.1097/AOG.0b013e31820ce2db

[16] Henriksson P, Westerlund E, Wallen H, Brandt L, Hovatta O, et al (2013) Incidence of pulmonary and venous thromboembolism in pregnancies after in vitro fertilisation: Cross sectional study. BMJ 346: e8632. 23321489 10.1136/bmj.e8632 PMC3546085

[17] Danilenko-Dixon DR, Heit JA, Silverstein MD, Yawn BP, Petterson TM, et al (2001) Risk factors for deep vein thrombosis and pulmonary embolism during pregnancy or post partum: A population-based, case-control study. Am J Obstet Gynecol 184: 104–110. 11174488 10.1067/mob.2001.107919

[18] Simpson EL, Lawrenson RA, Nightingale AL, Farmer RD (2001) Venous thromboembolism in pregnancy and the puerperium: Incidence and additional risk factors from a london perinatal database. BJOG 108: 56–60. 11213005 10.1111/j.1471-0528.2001.00004.x

[19] James AH, Jamison MG, Brancazio LR, Myers ER (2006) Venous thromboembolism during pregnancy and the postpartum period: Incidence, risk factors, and mortality. Am J Obstet Gynecol 194: 1311–1315. 16647915 10.1016/j.ajog.2005.11.008

[20] Larsen TB, Sorensen HT, Gislum M, Johnsen SP (2007) Maternal smoking, obesity, and risk of venous thromboembolism during pregnancy and the puerperium: A population-based nested case-control study. Thromb Res 120: 505–509. 17257657 10.1016/j.thromres.2006.12.003

[21] Jacobsen AF, Skjeldestad FE, Sandset PM (2008) Ante- and postnatal risk factors of venous thrombosis: A hospital-based case-control study. J Thromb Haemost 6: 905–912. 18363820 10.1111/j.1538-7836.2008.02961.x

[22] Sultan AA, Tata LJ, West J, Fiaschi L, Fleming KM, et al (2013) Risk factors for first venous thromboembolism around pregnancy: A population-based cohort study from the united kingdom. Blood 121: 3953–3961. 23550034 10.1182/blood-2012-11-469551

[23] Virkus RA, Lokkegaard EC, Lidegaard O, Langhoff-Roos J, Bjerregaard L, et al (2013) Venous thromboembolism in pregnancy and the puerperal period: A study of 1210 events. Acta Obstet Gynecol Scand 92: 1135–1142. 23869667 10.1111/aogs.12223

[24] Rothman KJ (1981) Induction and latent periods. Am J Epidemiol 114: 253–259. 7304560 10.1093/oxfordjournals.aje.a113189

[25] Prasad A, Zhu J, Halcox JP, Waclawiw MA, Epstein SE, et al (2002) Predisposition to atherosclerosis by infections: Role of endothelial dysfunction. Circulation 106: 184–190. 12105156 10.1161/01.cir.0000021125.83697.21

[26] Jensen TB, Gerds TA, Gron R, Bretler DM, Schmiegelow MD et al (2013) Risk factors for venous thromboembolism during pregnancy. Pharmacoepidemiol Drug Saf. 10.1002/pds.3536 24130063

[27] Carr MH, Towers CV, Eastenson AR, Pircon RA, Iriye BK, et al (1997) Prolonged bedrest during pregnancy: Does the risk of deep vein thrombosis warrant the use of routine heparin prophylaxis? J Matern Fetal Med 6: 264–267. 9360183 10.1002/(SICI)1520-6661(199709/10)6:5<264::AID-MFM4>3.0.CO;2-E

[28] Kovacevich GJ, Gaich SA, Lavin JP, Hopkins MP, Crane SS, et al (2000) The prevalence of thromboembolic events among women with extended bed rest prescribed as part of the treatment for premature labor or preterm premature rupture of membranes. Am J Obstet Gynecol 182: 1089–1092. 10819836 10.1067/mob.2000.105405

[29] Sultan A, West J, Tata L, Fleming K, Nelson-Piercy C et al (2013) Risk of first venous thromboembolism in pregnant women in hospital: Population based cohort study from england. BMJ 347. 10.1136/bmj.f6099 PMC3898207 24201164

[30] Ros HS, Lichtenstein P, Bellocco R, Petersson G, Cnattingius S (2002) Pulmonary embolism and stroke in relation to pregnancy: How can high-risk women be identified? Am J Obstet Gynecol 186: 198–203. 11854635 10.1067/mob.2002.119177

[31] Knight M (2008) UKOSS (2008) Antenatal pulmonary embolism: Risk factors, management and outcomes. BJOG 115: 453–461. 18201281 10.1111/j.1471-0528.2007.01622.x

[32] Robinson HE, O'Connell CM, Joseph KS, McLeod NL (2005) Maternal outcomes in pregnancies complicated by obesity. Obstet Gynecol 106: 1357–1364. 16319263 10.1097/01.AOG.0000188387.88032.41

[33] Liu S, Liston RM, Joseph KS, Heaman M, Sauve R, et al (2007) Maternal mortality and severe morbidity associated with low-risk planned cesarean delivery versus planned vaginal delivery at term. CMAJ 176: 455–460. 17296957 10.1503/cmaj.060870 PMC1800583

